# Cell Technologies in Treating Osteochondral Lesions of the Talus: A Clinical Case and Brief Review

**DOI:** 10.3390/jcm14227917

**Published:** 2025-11-08

**Authors:** Dina Saginova, Meruyert Makhmetova, Yerik Raimagambetov, Bagdat Balbossynov, Assel Issabekova, Lyudmila Spichak, Vyacheslav Ogay

**Affiliations:** 1 National Scientific Center of Traumatology and Orthopedics named after Academician N. D. Batpenov, Astana 010000, Kazakhstan; 2 Research School, Astana Medical University, Astana 010000, Kazakhstan; 3 National Center for Biotechnology, Astana 010000, Kazakhstan

**Keywords:** osteochondral lesion, talus, heparin-conjugated fibrin hydrogel, mesenchymal stem cells, arthroscopy

## Abstract

Osteochondral lesions of the talus (OLTs) present a significant clinical challenge, often leading to pain, dysfunction, and joint degeneration. Traditional treatments, including microfracture and grafting, have limitations in their ability to fully restore osteochondral integrity. Recent advances in tissue engineering have introduced heparin-conjugated fibrin hydrogel (HCFH) as a promising scaffold for regenerative therapy. By supporting mesenchymal stem cell (MSC) proliferation and controlled growth factor release, HCFH enhances cartilage and bone repair. A 21-year-old female presented with chronic right ankle pain and instability following a sports injury, with MRI revealing an osteochondral lesion in the lateral dome of the talus and an anterior talofibular ligament injury. Treatment included autologous MSC isolation, HCFH synthesis, arthroscopic debridement, microfracture, and implantation of MSC-loaded HCFH, while postoperative rehabilitation involved four weeks of restricted weight-bearing- and physiotherapy. At 12 months, her visual analog scale (VAS) score decreased from 60 to 40, indicating clinical improvement, and her American Orthopaedic Foot and Ankle Society (AOFAS) score increased from 69 to 77. Serial MRI scans showed progressive cartilage regeneration with near-complete defect filling. This case highlights the potential of MSC-loaded HCFH in treating OLTs. The observed improvements in pain relief, function, and cartilage regeneration suggest that this technique may overcome the limitations of conventional treatments. Further studies with larger cohorts and long-term follow-up are necessary to confirm its clinical efficacy.

## 1. Introduction

An osteochondral lesion of the talus (OLT) is a significant clinical problem characterized by damage to both the articular cartilage and subchondral bone, often resulting from trauma, ischemia, or degenerative joint diseases [1] and leading to severe pain, impaired function, and progressive osteoarthritis if left untreated [2,3]. Various treatment options have been explored, ranging from conservative management [4] to surgical interventions, including microfracture [5,6], autologous chondrocyte implantation (ACI) [7,8], and osteochondral autografts [9] or allografts. Despite these approaches, restoring the damaged cartilage and underlying bone remains suboptimal due to the avascular nature of the cartilage and the osteochondral unit’s complex regeneration process [10].

The introduction of tissue engineering and regenerative medicine has created new avenues for treating osteochondral lesions. Hydrogels, in particular, have gained attention for their ability to provide a supportive environment for cell proliferation and differentiation, mimicking the extracellular matrix (ECM) of natural cartilage [11,12]. They can be bioengineered to include various bioactive molecules and cells, enhancing their therapeutic potential. One promising approach is the use of fibrin-based hydrogels, which have inherent biocompatibility and the ability to degrade in a controlled manner, supporting tissue regeneration. Their use in various tissue engineering applications has been explored due to their role in mimicking the natural ECM and promoting cellular infiltration and tissue repair [13].

In recent years, the combination of hydrogels with mesenchymal stem cells (MSCs) has emerged as a powerful strategy to treat osteochondral lesions [14]. MSCs have been widely studied for their ability to differentiate into chondrocytes and osteoblasts, making them an ideal candidate for osteochondral repair [15,16]. When combined with hydrogels, MSCs can be delivered directly to the defect site, where they contribute to tissue regeneration through both differentiation and paracrine signaling. Furthermore, MSCs have immunomodulatory properties, meaning that they can enhance the repair process by reducing inflammation and promoting a regenerative environment.

Heparin-conjugated fibrin hydrogels offer an additional advantage by incorporating heparin-binding growth factors, such as transforming growth factor-beta (TGF-β) and bone morphogenetic proteins (BMPs), which are critical for cartilage and bone regeneration [17,18]. The heparin conjugation allows for the sustained release of these growth factors at the defect site, thereby enhancing the regenerative potential of the hydrogel–MSC construct [14]. This bioengineering approach not only supports cellular proliferation and differentiation but also provides a structural scaffold for tissue regeneration, addressing the limitations of conventional treatments.

This clinical case report describes the application of a heparin-conjugated fibrin hydrogel loaded with MSCs to treat a challenging OLT. The case highlights the potential of this regenerative approach to promote both cartilage and bone repair, providing a novel therapeutic option for patients with OLTs. We discuss the clinical outcomes, the biological mechanisms underlying the regenerative process, and the potential for this approach to be translated into broader clinical practice.

## 2. Case Presentation

A 21-year-old female was admitted to our Orthopedic Department because of an OLT of the right ankle and an anterior talofibular ligament injury. The patient gave written informed consent for the publication of her clinical data.

The patient presented with complaints of pain and instability in the right ankle, which she had twisted approximately 18 months prior to presentation while playing tennis. Over time, her symptoms progressively worsened.

**Figure 1:**
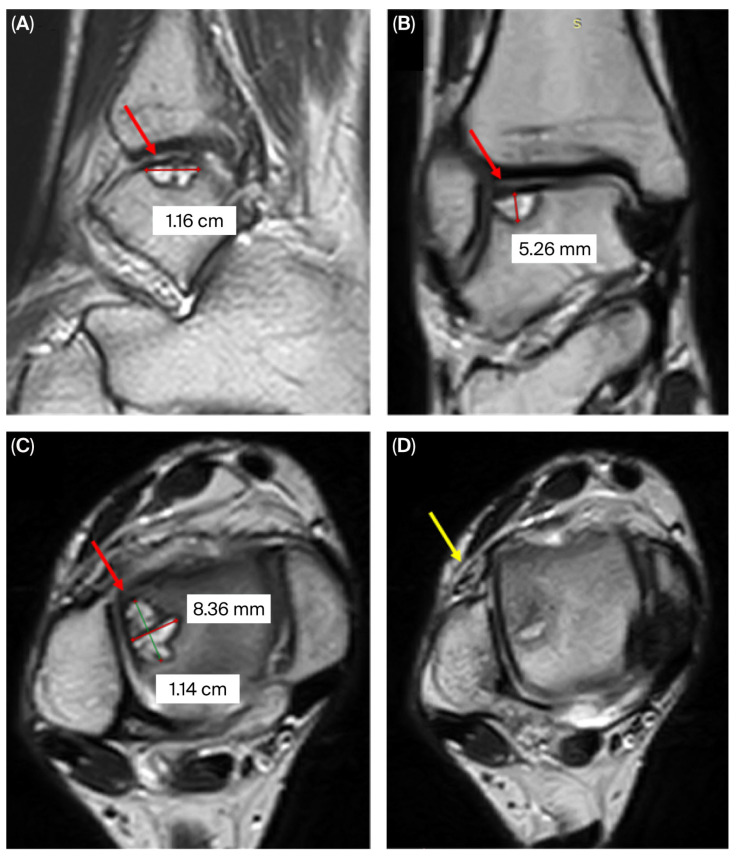
MRI of the right ankle joint showing the osteochondral lesion in the lateral talar dome (red arrow): (**A**) sagittal, (**B**) coronal, (**C**) axial; and an anterior talofibular ligament injury (yellow arrow) (**D**).

On physical examination, the patient was able to ambulate independently without assistive devices. The alignment of the lower extremities was normal, palpation revealed tenderness over the lateral dome of the talus, and the talar tilt test was positive. Magnetic resonance imaging (MRI) confirmed an OLT in the 6th quadrant [19] of the talus and an injury to the anterior talofibular ligament (Figure 1). After patient preparation, the first stage involved harvesting autologous mesenchymal stem cells and synthesizing a heparin-conjugated fibrin hydrogel (HCFH). The second stage included arthroscopy of the ankle joint with HCFH implantation.

**Stage 1—Synthesis of heparin-conjugated fibrin hydrogel from autologous adipose tissue mesenchymal stem cells and growth factors**

Microcannular tumescent liposuction

Klein’s solution consisting of 400 mL of saline, 20 mL of 2% lidocaine, 0.4 mL of epinephrine, and 10 mL of sodium bicarbonate was prepared. A total of 200 mL of the solution was infiltrated into the subcutaneous adipose tissue of the anterior abdominal wall to saturate the tissue and convert the fat cells into a liquid substance, which was then aspirated using a liposuction cannula. A total of 30 mL of subcutaneous adipose tissue was collected into syringes, which were then delivered to the stem cell laboratory of the National Center for Biotechnology (Astana, Kazakhstan) for MSC isolation.

Aspirated adipose tissue was rinsed three times with phosphate-buffered saline (PBS) and enzymatically digested in a 0.1% collagenase type I solution at 37 °C for 60 min under gentle agitation. Following digestion, the tissue was filtered through a 100-µm cell strainer (Corning, New York, NY, USA) to eliminate debris and then centrifuged at 300× *g* for 10 min to collect a cell pellet. The resulting pellet was resuspended in Minimum Essential Medium Eagle (Gibco, Waltham, MA, USA,) supplemented with 10% fetal bovine serum. The cell suspension was centrifuged at 300× *g* for 5 min and resuspended in MSC NutriStem^®^ XF culture medium (Sartorius AG, Goettingen, Lower Saxony, Germany) containing 1% antibiotic–antimycotic solution (Gibco, USA). The cell suspension was plated on a T25 cell culture flask (Corning, USA) for further cultivation at 5% CO_2_ and 37 °C. After 72 h, unattached cells were removed by thoroughly washing with PBS three times. When the monolayer reached 80% confluence, the cells were collected using TrypLE Express Enzyme (Gibco, Waltham, MA, USA) and subcultured into T75 cell culture flasks for expansion in MSC NutriStem^®^ XF culture medium, which was changed every 2 days.

In this study, all cultures of MSCs were expanded through passage 3. The culture-expanded MSCs were subsequently evaluated for cell count, viability, purity markers (CD31, CD34, CD45), identity-positive markers (CD73, CD90), sterility (bacterial, yeast, and fungal contamination), as well as bacterial endotoxin and mycoplasma contamination.

The main component of the gel was heparin-conjugated fibrinogen (HCF) [20]. The gel consisted of two components: component A and component B. To obtain the former, 40 mg/mL of HCF and 40 mg/mL of fibrinogen were dissolved in PBS (pH 7.4) at 37 °C for 1 h. Then, 2 × 10^7^ autologous adipose-derived MSCs were added to component A and gently mixed for 1 min. To obtain component B, 50 U/mL of thrombin, 250 U/mL of aprotinin, and 50 mM of CaCl_2_ were dissolved in PBS (pH 7.4). All components were sterilized by filtration through a polyethersulfone membrane filter with a pore size of 0.45%. For gelation, the freshly prepared components A and B were mixed in equal volumes, with a total gel volume of 3 mL. TGF-β and BMP-4 were added to the gel at a concentration of 1 µg/mL.

**Stage 2—Arthroscopic implantation of heparin-conjugated fibrin hydrogel**

**Figure 2:**
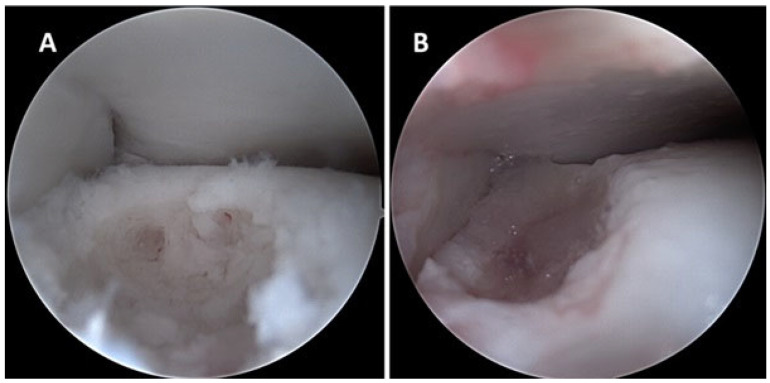
Ankle arthroscopy: (**A**) osteochondral defect of the talus; (**B**) filling the defect with HCFH.

Under spinal anesthesia, arthroscopic debridement of the right ankle joint was performed using an anterior approach. The OLT was cleared of non-viable tissue, and microfracturing was performed using an awl to further stimulate the subchondral bone. The defect was then filled via the Duploject System (Baxter International Inc., Deerfield, IL, USA) (Figure 2), achieving solidification within 10 min. The anterior talofibular ligament was subsequently repaired using two anchor sutures, following the Broström technique.

In the postoperative period, the patient’s right ankle was immobilized, and axial loading was restricted for four weeks. After four weeks, the patient began working with a rehabilitation specialist to restore ankle function.

**Figure 3:**
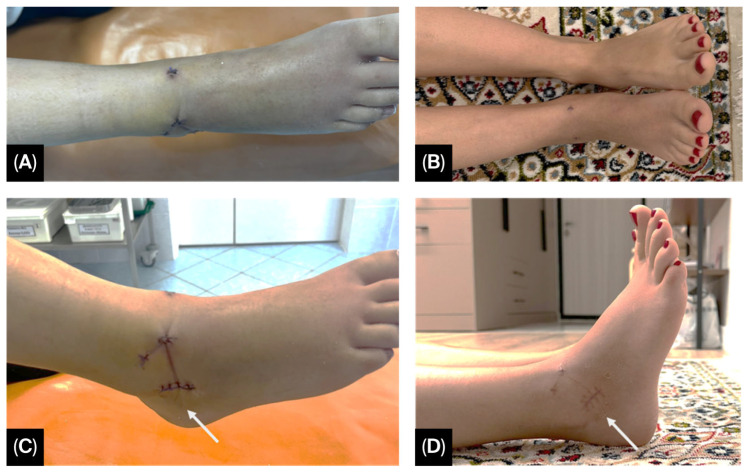
Condition of the patient’s right ankle joint on postoperative day 3: (**A**) anterior view; (**B**) lateral view, showing postoperative edema and moderate tension of the sutures due to swelling. One month after surgery: (**C**) anterior view; (**D**) lateral view, showing completely healed postoperative wounds and well-formed scars (arrow).

During the first postoperative week, the patient experienced swelling of the right foot, which subsided over time (Figure 3). The postoperative wounds showed no signs of infection and healed by primary intention.

Inflammation was evaluated by laboratory tests such as white blood cell count, erythrocyte sedimentation rate (ESR), and C-reactive protein (CRP). No changes were observed in the leukocyte count, so the preoperative leukocyte count was 5.61 × 10^9^/L, 7 days after surgery, it was 5.46 × 10^9^/L, and 14 days later, it was 4.57 × 10^9^/L (Table 1). There were also changes in ESR and CRP values; preoperatively, they were 11 Mm/h and 2.6 Mg/L, respectively. Seven days after surgery, these values increased to 24 Mm/h and 13 Mg/L, but after 14 days, the values were back within normal limits and were 15 Mm/h and 4.8 Mg/L, accordingly.

Functional assessment was performed using the AOFAS questionnaire and VAS, translated and validated in Kazakh [21], and osteochondral lesion regeneration was evaluated via MRI. The preoperative VAS score was 60, which decreased to 40, 12 months postoperatively, indicating a reduction in pain and an improvement in patient outcomes. The AOFAS score increased from 69 preoperatively to 77 at the 12-month follow-up, indicating improved functional outcomes due to the elimination of instability.

**Figure 4:**
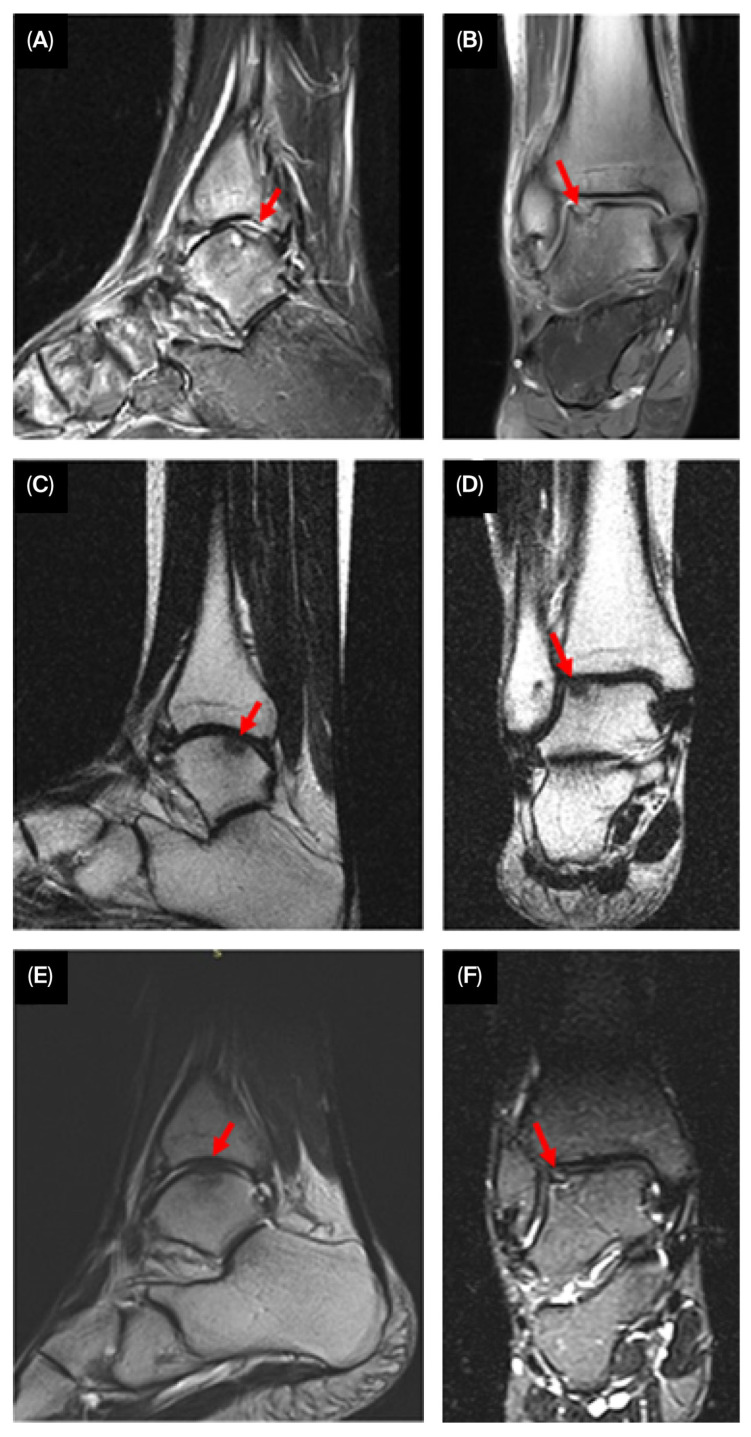
Serial MRI images showing progressive cartilage regeneration (arrowheads) and defect filling over 3 ((**A**)—sagittal, (**B**)—coronal), 6 ((**C**)—sagittal, (**D**)—coronal), and 12 months ((**E**)—sagittal, (**F**)—coronal).

Follow-up MRI examinations were performed 3, 6, and 12 months after treatment (Figure 4). Post-treatment coronal and sagittal MRI images indicate progressive regeneration of the articular cartilage at the site of the chondral defect, with smooth integration into the surrounding joint surface.

Semi-quantitative measurements of the cartilage defect were obtained using the modified International Cartilage Repair Society (ICRS) scoring system. Structural assessment via MRI demonstrated significant improvement, with near-complete filling of the OLT. The modified ICRS score improved from grade 3 to grade 1, indicating smooth integration between the newly formed cartilage and the surrounding native cartilage.

## 3. Discussion

The use of heparin-conjugated fibrin hydrogel to treat OLTs presents a novel and promising approach that addresses some of the limitations associated with traditional treatments. This discussion reflects on the advantages, clinical implications, and limitations of this emerging technique while integrating findings from various related studies.

Especially in weight-bearing joints like the talus, treating OLTs is challenging due to the complex structure and limited regenerative capacity of cartilage [10,22]. Traditional methods yield inconsistent long-term outcomes. Specifically, bone marrow stimulation (BMS) often leads to the formation of fibrocartilage instead of hyaline cartilage, which lacks the mechanical durability necessary for sustained support [23,24]. In osteochondral autograft transplantation (OAT), achieving complete congruence of the articular surfaces is critical; Latt LD et al. found that a 1.0 mm protrusion of the graft significantly increased the pressure on it [25]. Autologous chondrocyte implantation (ACI) involves a two-step procedure, which increases its cost. Additionally, harvesting cartilage from the donor site heightens the risk of trauma and inflammation in the donor joint. Giannini S et al. reported cases of hypertrophy of the transplanted cartilage, the presence of subchondral pseudocysts, and subchondral edema in a 10-year follow-up of 10 patients [8].

The incorporation of advanced biomaterials like heparin-conjugated fibrin hydrogel is intended to overcome these challenges by enhancing the delivery and retention of growth factors, as well as providing a scaffold for cellular proliferation and tissue regeneration.

The functionalization of fibrin hydrogels with cartilage extracellular matrix (ECM) components has been shown to significantly promote chondrogenesis, especially when combined with growth factors like TGF-β [13]. This case highlights the use of bioengineered hydrogels that promote both cartilage and subchondral bone repair. Heparin-conjugated hydrogels not only mimic the native ECM but also allow for sustained and controlled release of growth factors such as TGF-β and BMPs, which are crucial for osteochondral repair [12,14].

In combination with the inherent regenerative potential of MSCs, this approach appears to facilitate the regeneration of hyaline-like cartilage. Several studies have highlighted the potential of MSCs, especially adipose-derived mesenchymal stem cells (ADMSCs), in promoting cartilage repair and modulating the local inflammatory environment [26,27,28]. Studies have shown that the use of MSC-laden hydrogels can significantly improve the quality of the repaired cartilage, supporting the formation of hyaline-like tissue, which better withstands mechanical stress in weight-bearing joints like the talus [29]. The present case adds to this body of evidence by demonstrating successful integration of hyaline-like cartilage following the application of this dual-modality treatment. Moreover, fibrin-based scaffolds are well-tolerated and biodegrade in a manner that aligns with tissue healing, making them ideal for long-term tissue support [30,31].

Despite the promising results observed in this case, challenges remain. The variability in outcomes associated with MSC-based therapies has been well-documented, with some studies reporting inconsistent long-term results [26,31]. Factors such as the source of MSCs, the quality of the scaffold material, and the mechanical environment of the joint all play critical roles in determining the success of cartilage repair. It is notable that ADMSCs have been shown to possess lower chondrogenic potential compared to bone marrow-derived MSCs (BMMSCs) in some comparative studies [28,31]; however, the ease of harvesting ADMSCs, along with their potent immunomodulatory and anti-inflammatory effects, makes them an attractive option for cartilage repair [27,28].

Previous experimental studies have highlighted the regenerative potential of heparin-conjugated fibrin hydrogels as delivery systems for osteoinductive growth factors, particularly members of the bone morphogenetic protein (BMP) family. For example, a study using a heparin-conjugated fibrin hydrogel incorporating bone morphogenetic protein-2 (BMP-2) and adipose-derived pericytes demonstrated enhanced osteogenesis and accelerated healing of critical-sized calvarial defects in rats [32]. Similarly, Almeida et al. [13] reported that fibrin hydrogels functionalized with extracellular matrix (ECM) components supported chondrogenic differentiation, especially when combined with exogenous growth factors.

Building on these findings, our group conducted preclinical research evaluating a heparin-conjugated fibrin hydrogel incorporating mesenchymal stem cells (MSCs), transforming growth factor-β (TGF-β), and BMP-4. This study demonstrated that the hydrogel provided a biocompatible matrix enabling sustained release of TGF-β1 and BMP-4, and that co-delivery of autologous MSCs with these growth factors significantly enhanced osteochondral regeneration, resulting in complete restoration of hyaline cartilage and subchondral bone in vivo [20].

Despite these promising results, the use of these hydrogels is not without its challenges. The biocompatibility, degradation rate, and mechanical properties of the scaffold must be carefully balanced to match the requirements of the joint environment [20]. Furthermore, while initial clinical outcomes are encouraging, long-term studies are necessary to confirm the durability of the repair and to assess potential complications such as graft failure or inflammation. Additionally, the cost and technical complexity of producing such bioengineered scaffolds may limit their immediate widespread use [33].

One of the main limitations of this study is that it presents only a single clinical case, which restricts our ability to draw generalizable conclusions. However, this clinical case was conducted as part of a scientific project (Grant No. AP19679620) aimed at exploring regenerative technologies for osteochondral repair, so in the future, we plan to conduct a larger-scale clinical study involving a broader patient cohort with similar lesion characteristics. The aim of this upcoming research is to evaluate the reproducibility, long-term effectiveness, and safety of the technique, as well as to compare its outcomes with those of conventional conservative approaches.

Moreover, several challenges may hinder the widespread adoption of this method in clinical practice: the necessity for GMP-compliant laboratory infrastructure, the availability of skilled personnel in regenerative medicine, and the relatively high cost of cell-based therapies. Such factors can limit the feasibility of implementing this technique in smaller or non-specialized medical institutions. Addressing these barriers will be crucial for enabling broader clinical translation of this method.

Additionally, the relatively short follow-up period limits our ability to assess the treatment’s long-term outcomes and durability. Further studies involving larger sample sizes and longer observation periods are necessary to validate these findings and provide more comprehensive insights.

## 4. Conclusions

The application of heparin-conjugated fibrin hydrogel in the treatment of osteochondral lesions represents a significant advancement in cartilage repair. By providing a bioactive scaffold that supports natural healing, this approach may overcome the main limitation of conventional therapies—the formation of fibrocartilage with poor mechanical strength. However, given the lack of histological or biochemical validation and the limited sample size, these findings should be considered preliminary. Further studies involving larger patient cohorts and detailed biological analyses are required to confirm the therapeutic potential of this approach.

## Figures and Tables

**Table 1:** Changes in laboratory and functional parameters before and after surgery.

	Leu (×10^9^/L)	ESR (mm/h)	CRP (mg/L)	VAS	AOFAS
Before surgery	5.61	11	2.60	60	69
1 week after surgery	5.46	24	13	-	-
4 weeks after surgery	4.57	15	4.80	-	-
6 months after surgery	-	-	-	55	74
12 months after surgery	-	-	-	40	77

Leu—leukocytes; ESR—erythrocyte sedimentation rate; CRP—C-reactive protein; VAS—visual analog scale (pain); AOFAS—American Orthopaedic Foot and Ankle Society score.

## Data Availability

The data of this study is available from the corresponding author, Meruyert Makhmetova, upon reasonable request.
